# Factors Associated With Delayed Contraceptive Implant Removal in Ethiopia

**DOI:** 10.9745/GHSP-D-20-00135

**Published:** 2020-09-30

**Authors:** Elizabeth Costenbader, Alice F. Cartwright, Misti McDowell, Berhane Assefa, Meza Yirga Tejeji, Eskindir Tenaw

**Affiliations:** a FHI 360, Durham, NC, USA.; b Department of Maternal and Child Health, Gillings School of Global Public Health, University of North Carolina at Chapel Hill, Chapel Hill, NC, USA.; c FHI 360-Ethiopia, Addis Ababa, Ethiopia.; d Federal Ministry of Health, Addis Ababa, Ethiopia.

## Abstract

**Background:**

In 2009, the Government of Ethiopia initiated the implant scale-up initiative, which expanded contraceptive access by training health extension workers (HEWs) to insert single-rod etonogestrel contraceptive implants (Implanon) at rural health posts. Removals were provided by referrals to higher levels of the health system. However, little was known about whether women were getting their implants removed at the recommended 3-year postinsertion date or what barriers they faced to removal.

**Methods:**

Between June and July 2016, 1,860 Ethiopian women, who had a 1-rod etonogestrel implant inserted by either an HEW or another health care provider between 3 and 6 years prior, were surveyed. We describe the characteristics of the sample and use multivariable logistic regression to predict factors associated with keeping implants inserted beyond 3 years.

**Results:**

Women who had received their implants from HEWs were significantly more likely to report keeping them inserted for more than 3 years (adjusted odds ratio=2.50; 95% confidence interval=1.19, 5.24), compared with those who got their implant from another health care provider. Women who reported distance to the facility or transportation as a barrier were also significantly more likely to keep their implant for more than 3 years. Married and educated women were less likely to keep their implants for an extended duration. Among women who had their implant for 3 years or less, women who had had it inserted by an HEW were significantly more likely to report that the provider was unable or refused to provide removal as a barrier.

**Discussion:**

Efforts to expand lower level and community-based access to contraceptive implants that do not ensure reliable access to removals at the same level as insertions may lead to women using implants beyond the recommended duration.

## Abstract (precis)

Women receiving implant insertion at the community level were significantly more likely to report keeping their implant for more than 3 years. Even when a referral or back-up system for removals existed, efforts to task-shift the provision of contraceptive implants may have inadvertently led to extended implant use.

## Teaser

**Key Findings:**

- Women receiving implant insertion at the community level were significantly more likely to report keeping their implant for more than 3 years.
- Task sharing only implant insertion and not removal services to community-based health worker cadres may have inadvertently led to extended implant use, particularly in rural areas or areas far from facilities.

**Key Implications:**

- Governments and program planners should prioritize efforts to ensure that implant removal services are available at the same health system level as insertions.
- National stakeholders and implementing partners should consider including implant removal as part of the training for all health care providers who are trained in insertion.

## INTRODUCTION

Although Ethiopia has made considerable progress in increasing access to modern contraception over the past few decades,^1^ Ethiopian women still have a high level of unmet need for contraception (20.6% for married women and 13.9% for all women), especially in rural areas (22.6% for rural married women compared with 13.1% in urban areas).^2^ Part of the high unmet need among women in rural areas of Ethiopia may be driven by lack of access to facility-based health care in these areas. In 2009, to increase the national contraceptive prevalence and reduce unmet need for contraception, the Ethiopian Federal Ministry of Health (FMOH) launched an implant scale-up initiative to expand the family planning method mix and increase voluntary access to long-acting reversible contraceptives (LARCs).^3^^,^^4^ To achieve this goal, the FMOH made Implanon, a subdermal single-rod progestin-only implant,^5^^,^^6^ available at rural health posts by training health extension workers (HEWs) in its provision. Although clinical studies have found that 1-rod etonogestrel contraceptive implants may be effective for up to 5 years,^7^ since coming onto the market, Implanon (and its current analogue, Implanon NXT or Nexplanon) have only been approved for 3 years of effective use for pregnancy prevention before needing removal.^5^

HEWs are government employees who are trained to provide a variety of services to rural communities. As part of the national Health Extension Program, which was launched in 2003 to increase access to preventive and curative health care services, HEWs provide services either at rural health posts or in residents’ homes. These preventive and curative services fall into 4 broad areas: health education and communication, hygiene and environmental sanitation, disease prevention and control, and family health services, which include family planning and adolescent reproductive health packages.^8^ HEWs counsel on the full range of methods and provide pills, condoms, and injectable contraceptives.^3^^,^^9^

Before 2009, HEWs referred women who wished to obtain LARCs to a higher level of the health system. As part of the implant scale-up initiative, the FMOH collaborated with partner organizations to train HEWs in implant insertion.^4^ Notably, only the 1-rod contraceptive implant insertion was approved for task sharing to the HEW cadre. Substantial effort was also dedicated toward demand creation and ensuring the logistical commodity supply to expand accessibility. Currently, Implanon is the most widely used implant in Ethiopia and the most commonly used LARC method.^10^^–^^12^ In the first 6 years of the scale-up in Ethiopia (2009–2015), more than 1.2 million single-rod etonogestrel contraceptive implant insertions occurred. During the same time period, only 37,175 Implanon removals were documented.^4^

HEWs were trained to counsel women on where to access removal services if they wanted the 1-rod contraceptive implant removed. Because implant removal involved making an incision, it was determined to be outside of the HEWs’ scope of practice, thus they were not trained in implant removal. Instead, as part of the implant scale-up initiative, additional health care providers at higher-level facilities were trained in removals, and mobile teams of these providers were sent to health posts and the community to provide re-movals on a periodic basis.^4^^,^^13^ Nonetheless, given the large number of implants provided by HEWs at the community level since the initiation of the implant scale-up and the relatively few documented removals, information is limited regarding whether single-rod contraceptive implant users were able to get their implants removed at the recommended 3-year postinsertion date (or at any other time that they may have desired). Further, the barriers to removal that they may have faced are unknown.

For this study, we used data collected in 2016 to inform future planning of family planning service provision in Ethiopia and to assess whether women who received 1-rod contraceptive implants since the inception of the implant scale-up initiative had experienced any barriers to removal. The original parent study was further designed to inform reco-mmendations to improve contraceptive implant service delivery. The main objectives of the current analysis were to test the hypothesis that single-rod contraceptive implant insertion by an HEW would be associated with keeping the implant for longer than 3 years and to assess which factors were most strongly associated with keeping implants inserted for longer than 3 years.

We wanted to assess whether having a 1-rod implant inserted by an HEW would be associated with keeping the implant longer than 3 years and what factors were associated with keeping it longer.

## METHODS

### Study Design and Sample

The data used for this analysis are from a survey conducted in the 4 regions in which the implant scale-up initiative with HEWs initially took place: Amhara; Oromia; Tigray; and Southern Nations, Nationalities, and Peoples’ Region (SNNPR). Data collection for the survey took place between June and July 2016 at 160 health posts and 40 health centers across those 4 regions.

### Sampling Design

A stratified 3-stage cluster sample design was used to select survey respondents: woredas (districts), health facilities, and implant users were chosen as the first, second, and third stages of sampling, respectively. Before their selection, the woredas were stratified by urban and rural classifications. Subsequently, based on sample size power calculations, we determined that 12 woredas per region (for a total of 48 woredas) and 4 or 5 health facilities per woreda would be needed to reach our target of 200 health facilities and 2,000 women distributed equally across the 4 regions and proportionately across urban and rural strata.

We obtained lists of woredas from the Central Statistical Agency of Ethiopia within each of our 4 study regions, along with the estimated number of women of reproductive age. From each of these woreda health offices, we obtained lists of all their health facilities along with lists of the estimated number of 1-rod contraceptive implant users of reproductive age that were associated with each health facility. From these lists, we used a probability proportional to size sample selection scheme, taking the number of women of reproductive age within a woreda as a measure of size, to select woredas. The same sample selection scheme was used in selecting health facilities within woredas, using the number of single-rod contraceptive implant users as the measure of size. Finally, from the sampled health facilities, we obtained anonymized lists of implant users (who currently have or had a 1-rod contraceptive implant inserted between 3 and 6 years before the date of the interview). From these lists, contraceptive implant users were then chosen using equal probability systematic sample selection procedures.

### Survey Procedures

The women eligible to be sampled for this study were identified in each health facility (health post and health center) from the family planning registers and family folders. These records were only accessed by clinic employees. The clinic employees first generated a list of all women who met the study eligibility criteria: willing and able to give informed consent for participation, age between 18 and 49, living in the catchment area of the health facility, and had a 1-rod etonogestrel contraceptive implant inserted between 3 and 6 years before the date of interview, along with an associated client identification number. An anonymized list of client identification numbers was shared with the study data collectors to sample a subset of women to be interviewed.

After a woman was selected, a health provider contacted each woman and requested that she come to the health post or health center on a specific day for the interview. Because the majority of women did not have phones, HEWs contacted the selected women in person either at the health facility or at the woman’s home. Once in a private location, the HEW described the study in general terms and asked if the woman was interested in learning more about the study. For those who were interested, HEWs provided additional details about the study to confirm eligibility and gave the women a date and time to come to the health post for study enrollment and to take part in an interview. Upon arrival at the health post, members of the study team described the study, reconfirmed eligibility, obtained informed consent, and administered the survey.

The survey tool included approximately 36 questions pertaining to participant demographics, participant contraceptive implant use (including when and by whom the implant was inserted, what information was provided by the provider on contraceptive implant use and removal, whether the implant was removed and if so, when, by whom, and the reason for removal, and any barriers/challenges to removal), and any subsequent/current family planning use. If a woman had had more than 1 implant inserted during the 3- to 6-year time frame, the interviewer was instructed to ask her about the latest implant. The interviews took place in a private room at the health facility, and if no private room was available, a place outside was used where they could not be overheard. The survey was paper based; conducted in either Amharic, Oromifa, or Tigrigna language, as applicable; and lasted approximately 20 minutes. Participants were reimbursed 50 birr (US$2.40) for their travel to and from the health facility for the interview. This study was reviewed and approved by the institutional review boards at the Ethiopian Public Health Institute and FHI 360 (Protection of Human Subjects Committee) in May 2016.

### Analysis

The survey data were entered into EpiData 3.1 and then exported to SAS for analysis. To reduce biases introduced by the lack of reliable sampling frames and interviews refused or lost by primary sampling unit and strata, we constructed sampling weights to apply to the survey data. Key outcome indicators in the data were reviewed using bivariate analyses, including chi-square tests for significance. We then tested the bivariate relationship between keeping an implant beyond the recommended timeframe (3 years after insertion) and selected independent variables that we expected to affect implant removal in Ethiopia using design-adjusted chi-square tests. Based on the efforts to train HEWs to provide 1-rod contraceptive implant insertions but not removals, we hypothesized *a priori* that implant insertion by an HEW would be associated with keeping the implant for longer than 3 years. Informed by the results of the bivariate analyses, we subsequently developed a multivariable logistic model, including interactions between sociodemographic characteristics and the type of provider that inserted the implant. We ultimately selected a model based on model fit and collinearity assessments.

Based on the efforts to train HEWs to provide implant insertions but not removals, we hypothesized a priori that implant insertion by an HEW would be associated with keeping the implant for longer than 3 years.

## RESULTS

**FIGURE 1.**
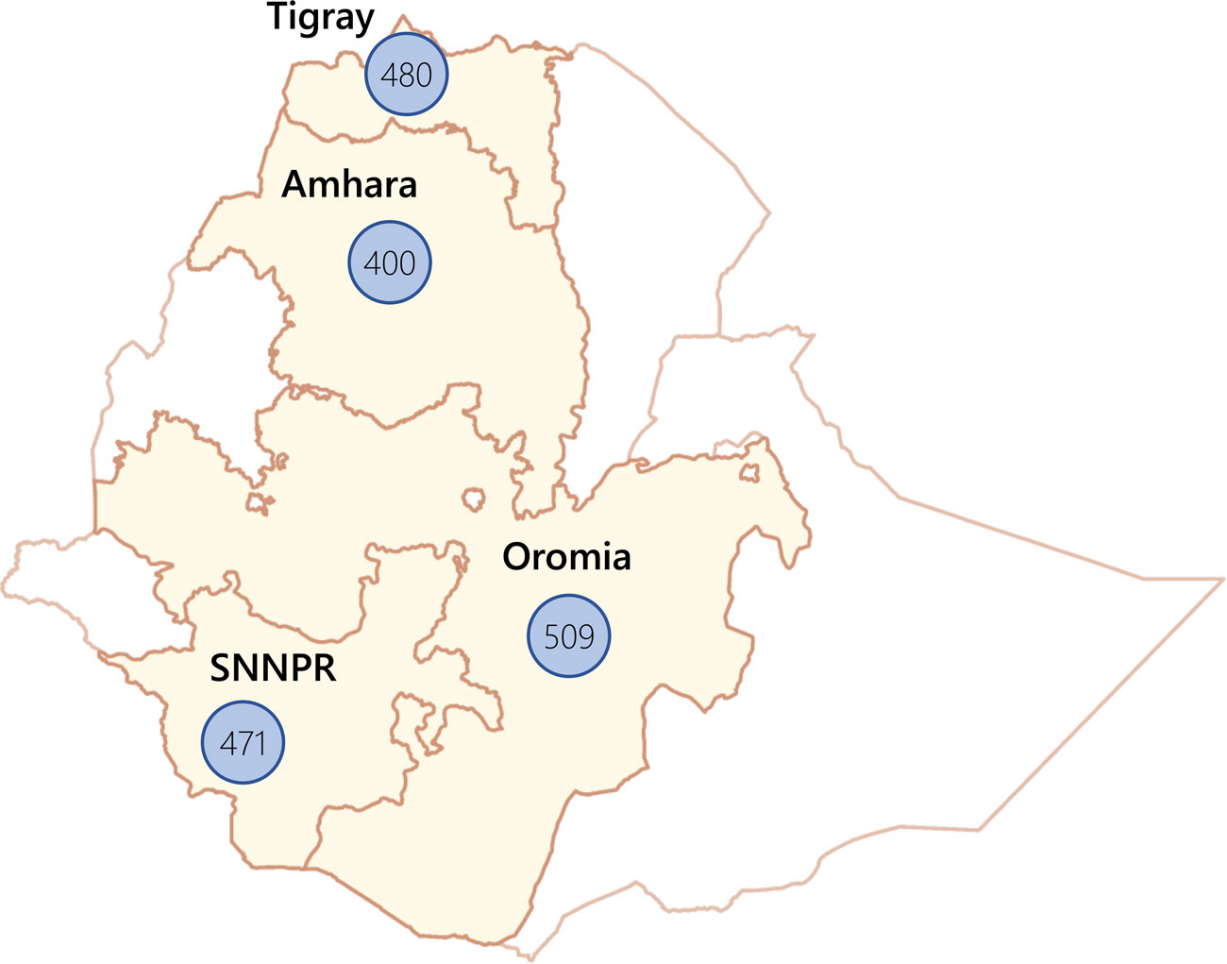
Number of Implanon Users Surveyed in Ethiopia, by Region Abbreviation: SNNPR, Southern Nations, Nationalities, and Peoples’ Region.

A total of 1,860 Ethiopian single-rod contraceptive implant users completed the survey (Figure 1). Notably, due to the security issues in Amhara during data collection, only 10 woredas were sampled instead of the planned number of 12. Table 1 shows the sociodemographic characteristics of contraceptive implant users by the provider that originally inserted their implant. Overall, women who had their implant inserted by an HEW were slightly older, significantly more likely to report having no education, to be married, and to live in Amhara or SNNPR compared with women surveyed who had had their implant inserted by another health provider at a health center. Those women whose 1-rod contraceptive implant had originally been inserted by an HEW were also significantly more likely to report no current contraceptive use and significantly less likely to report current use of a contraceptive implant. There was no significant difference in reasons provided for getting the contraceptive implant inserted (e.g., for spacing or limiting pregnancies) between those who had received the implant from an HEW versus another health provider (results not shown).

**TABLE 1.** Demographic Characteristics of Study Participants by Provider That Inserted Implanon^a^

	HEW (n=1,346)	Other Health Provider (n=514)	Total (N=1,860)	*P*-value
**Age** ^**b**^				.0017
18–24	166 (12.3)	86 (18.0)	252 (14.4)	
25–34	704 (57.3)	283 (50.7)	987 (54.8)	
35–44	397 (27.2)	133 (30.5)	530 (28.5)	
45–49	76 (3.2)	12 (0.8)	88 (2.3)	
**Level of education**				.0012
No education	807 (60.3)	214 (46.0)	1,021 (55.0)	
Read and write	82 (6.2)	25 (4.7)	107 (5.6)	
Primary	374 (28.2)	187 (38.9)	561 (32.2)	
Secondary	80 (5.0)	71 (8.6)	151 (6.4)	
Tertiary	3 (0.3)	17 (1.8)	20 (0.8)	
**Religion** ^c^				.84
Orthodox	712 (42.0)	244 (36.2)	956 (39.8)	
Protestant	328 (37.7)	123 (36.8)	451 (37.4)	
Muslim	276 (18.5)	136 (23.7)	412 (20.5)	
Catholic/other	30 (1.8)	11 (3.3)	41 (2.4)	
**Marital status**				<.001
Married	1,219 (93.1)	443 (85.7)	1,662 (90.3)	
Divorced/widowed/separated	110 (5.5)	59 (10.3)	169 (7.3)	
Never married	17 (1.4)	12 (4.0)	29 (2.4)	
**Employment status** ^**d**^				.42
Farm work	844 (54.7)	225 (67.3)	1,069 (59.4)	
Housewife	266 (27.1)	109 (14.0)	375 (22.2)	
Merchant	162 (10.9)	100 (10.9)	262 (10.9)	
Public servant/other/student/not employed	74 (7.3)	80 (7.9)	154 (7.5)	
**Region**				.0059
Tigray	379 (10.5)	101 (12.7)	480 (11.3)	
Oromia	274 (24.8)	235 (63.7)	509 (39.4)	
Amhara	304 (18.4)	96 (5.5)	400 (13.5)	
SNNPR	389 (46.4)	82 (18.1)	471 (35.8)	
**Contraceptive method(s) currently using** ^**e**^				
None	511 (39.7)	152 (20.1)	663 (32.3)	<.001
Implanon	332 (25.0)	176 (49.1)	508 (34.0)	<.001
Other implant	15 (2.0)	7 (1.8)	22 (1.9)	.94
Injectable	432 (28.8)	145 (24.3)	577 (27.1)	.53
Pills	24 (1.9)	23 (3.4)	47 (2.5)	.35
IUD	26 (2.1)	9 (1.1)	35 (1.8)	.36
Condom/calendar/other	7 (0.6)	4 (0.2)	11 (0.4)	.13

Abbreviations: HEW, health extension worker; IUD, intrauterine device; SNNPR, Southern Nations, Nationalities, and Peoples’ Region.

a Frequencies and weighted percentages reported.

b Three people missing age (all in HEW group).

c Other religion includes Traditional religion (“Wakefeta”) and “Jesus only” (“Hawariyat”).

d Other employment includes day laborer, construction worker, maid/janitor, and self-employed.

e Respondents could choose more than one method of family planning. Other methods include Sino-implant and female sterilization.

Although women who had their single-rod contraceptive implant inserted by an HEW were equally likely as women who had their implant inserted by another health care provider to report being informed about possible side effects of the implant and when it should be removed, they were slightly, but significantly more likely to report that they could not remember if they were told where to get their implant removed (Table 2). However, the overwhelming majority of women in both groups reported having been told when their implant should be removed (95% and 96%, respectively) as well as advised where they could get it removed (93% and 93%, respectively), and just over three-quarters (76% and 77%, respectively) reported being informed about possible side effects of a 1-rod contraceptive implant. In terms of duration of use of the contraceptive implant, over one-quarter (26%) of women who had had their implant inserted by an HEW reported using it for more than 3 years (21%) or that it was still inserted (3–6 years after insertion) (5%), compared with just over 10% (11% more than 3 years and 1% still inserted) of those who had had implant insertion performed by another provider, a statistically significant difference.

**TABLE 2.** Characteristics of Implanon Insertion Experience and Use by Provider That Inserted Implanon^a^

	HEW (n=1,346)	Other Health Provider (n=514)	Total (N=1,860)	*P*-value
**Informed about possible side effects of Implanon**				.61
Yes	1,026 (76.8)	404 (77.3)	1,430 (77.0)	
No	300 (21.9)	105 (22.2)	405 (22.0)	
Can’t remember/no response	20 (1.3)	5 (0.5)	25 (1.0)	
**Told when Implanon should be removed**				.46
Yes	1,276 (95.0)	490 (96.5)	1,766 (95.6)	
No	61 (4.1)	21 (3.2)	82 (3.7)	
Can’t remember/no response	9 (0.9)	3 (0.3)	12 (0.7)	
**Advised where they could get Implanon removed**				.021
Yes	1,230 (93.0)	482 (93.1)	1,712 (93.1)	
No	105 (6.2)	31 (6.9)	136 (6.5)	
Can’t remember/no response	11 (0.7)	1 (0.0)	12 (0.4)	
**Duration of Implanon use** ^**b**^				.0010
<1 year	30 (1.7)	19 (2.0)	49 (1.8)	
1 year to <3 years	251 (16.7)	100 (14.7)	351 (16.0)	
3 years	781 (55.3)	311 (70.9)	1092 (61.1)	
>3 years^c^	284 (26.3)	84 (12.4)	368 (21.1)	

Abbreviation: HEW, health extension worker.

a Frequencies and weighted percentages reported.

b Includes 2 respondents who said their implant was missing.

c Includes 92 respondents who still had their implant inserted at the time of interview.

Five percent of survey respondents reported still having their contraceptive implant at the time of the survey, although it was past the recommended removal date (Table 2 footnote). The calculated days past recommended removal ranged from 5 to 1,377 days. These women gave various reasons for not having had their implant removed, including they were planning on getting it removed soon (23%), did not know the removal date (22%), faced barriers to getting it removed (14%), and miscellaneous other reasons (41%) (results not shown).

In the multivariable logistic regression analyses, respondents who had their 1-rod contraceptive implant inserted by an HEW had 2.5 times the odds of keeping the implant for more than 3 years (adjusted odds ratio [aOR]=2.50; 95% confidence interval [CI]=1.19, 5.24) (Table 3). Living in SNNPR compared with Tigray (aOR=2.74; 95% CI=1.40, 5.35) or reporting distance or transportation to the facility as a barrier to removal (aOR=3.31; 95% CI=1.61, 6.78) were also factors associated with keeping the implant for longer than 3 years. Women who were literate/had any education (aOR=0.73; 95% CI=0.54, 1.00) and those who were married (aOR=0.55; 95% CI=0.39, 0.79) were significantly less likely to have kept their implant beyond 3 years. When sociodemographic characteristics were interacted with provider who inserted the implant (Table 4), older women and women of other religions who had their implant inserted by an HEW were significantly more likely to have kept their implant for more than 3 years, compared with women in those same categories whose implant was inserted by another health provider.

**TABLE 3.** Multivariate Logistic Regression Model for the Association Between Health Provider Who Inserted Implant and Keeping Implant for Longer Than 3 Years^a^ (N=1,860)

	**OR (95% CI)**
**Health extension worker (ref: other health worker)**	2.50^c^ (1.19, 5.24)
**Client Age, years (ref: 25–34)**	
18–24	1.46 (0.58, 3.68)
35–44	1.20 (0.83, 1.72)
45–49	1.16 (0.58, 2.31)
**Literate/any education (ref: illiterate/no education)**	0.73^b^ (0.54, 1.00)
**Married (ref: unmarried)**	0.55^b^ (0.39, 0.79)
**Orthodox (ref: any other religion)**	1.07 (0.54, 2.12)
**Region (ref: Tigray)**	
SNNPR	2.74^c^ (1.40, 5.35)
Oromia	2.25 (0.86, 5.90)
Amhara	1.68 (0.64, 4.44)
**Reported distance to facility/transportation as a barrier**	3.31^c^ (1.61, 6.78)

Abbreviations: CI, confidence interval; OR, odds ratio; SNNPR, Southern Nations, Nationalities, and Peoples’ Region.

a This model does not include any interaction terms.

b *P*<.05

c *P*<.001

**TABLE 4.** Adjusted^a^ Odds Ratios Comparing Health Extension Workers and Other Health Workers for the Association Between Exposure Variable and Keeping Implant for 3 Years or Longer (N=1,860)

**Exposure**	**Adjusted OR**^b^^,^^c^ **(95% CI)**
**Age**	
18–24	0.65 (0.17, 2.53)
25–34	2.45 (0.82, 7.36)
35–44	6.17^d^ (2.31, 16.48)
45–49	0.74 (0.10, 5.97)
**Religion**	
Orthodox religion	0.87 (0.43, 1.78)
Any other religion	3.08^e^ (1.07, 8.82)

Abbreviations: CI, confidence interval; OR, odds ratio.

a Odds ratio adjusted for age, education, marital status, religion, region, and facility/transport barriers.

b Odds ratios shown for each subgroup to interpret significant interaction effects.

c Only covariates with significant interactions shown.

d *P*<.001.

e *P*<.05.

Respondents who had their 1-rod contraceptive implant inserted by an HEW had 2.5 times the odds of keeping the implant for more than 3 years.

Survey respondents also reported if they had experienced any barriers to accessing implant removal. Table 5 shows that among women who had their implant removed at or before 3 years, those who had their implant inserted by an HEW were significantly more likely to report experiencing barriers, including that the provider was unable or refused to provide removal or that they faced challenges in transportation or the distance to the facility. Among the women who had kept their single-rod contraceptive implant for longer than 3 years, there were no significant differences by provider who inserted the contraceptive implant in barriers reported to implant removal, although about one-third of these women said they forgot or did not know their removal date or that the provider was unavailable when they visited the facility.

**TABLE 5.** Barriers to Removal by Timing of Removal and by Provider That Inserted Implanon^a^

	**HEW**	**Other Health Provider**	**Total**	***P*-value**
**Removal at or before 3 years**	n=1,062	n=430	n=1,492	
No barriers	868 (83.9)	368 (90.0)	1,236 (86.4)	.029
Service provider unavailable day of visit	47 (3.9)	16 (3.7)	63 (3.8)	.88
Service provider unable to provide removal^b^	15 (1.8)	4 (0.2)	19 (1.1)	<.001
Service provider refused to provide removal	59 (5.7)	21 (2.4)	80 (4.3)	.015
Distance to facility/transportation	72 (4.8)	12 (2.1)	84 (3.6)	.020
Other barriers^c^	20 (1.9)	13 (0.9)	33 (2.0)	.90
**Removal more than 3 years after insertion/still inserted**	n=284	n=84	n=368	
No barriers	110 (38.7)	38 (34.6)	148 (37.8)	.61
Forgot or did not know removal date	89 (32.6)	29 (31.4)	118 (32.3)	.88
Service provider unavailable day of visit	80 (28.1)	15 (25.6)	95 (27.6)	.67
Service provider unable to provide removal^b^	18 (5.8)	5 (4.6)	23 (5.6)	.76
Service provider refused to provide removal	16 (3.8)	7 (6.2)	23 (4.3)	.39
Distance to facility/transportation	43 (11.8)	2 (9.9)	45 (11.4)	.83
Other barriers^c^	61 (26.7)	23 (32.9)	84 (28.1)	.50

Abbreviation: HEW, health extension worker.

a Frequencies and weighted percentages reported. Respondents could select more than one barrier.

b Includes lack of materials to provide removal.

c Other barriers include lack of time to go to facility, fear, and cost.

## DISCUSSION

Providing community-based health services in Ethiopia has helped the FMOH to significantly accelerate scale-up of LARCs, and training HEWs in single-rod etonogestrel contraceptive implant insertion is major part of this effort. However, our analysis demonstrates that alongside this increase in access, Ethiopian women have faced challenges to obtaining timely removals. In particular, women who received their implants from HEWs were significantly more likely to keep their implants inserted beyond the recommended 3-year timeframe. Research on the experience of introducing contraceptive implants in South Africa suggests that providers may perceive that women discontinue implants at a higher rate than other methods and feel that early removal is “wasteful” and therefore discourage it.^14^ In this study, we did not find high proportions of women keeping implants due to provider refusals to remove them. However, clients of HEWs were more likely to report provider refusals, so it is possible that HEWs refused to provide removals because they were not trained to do so.

It is also possible that human resource capacity was insufficient to meet the demand for removal services among participants who had their implants inserted by HEWs. Although health center staff were trained in implant removals, each health center is a referral site for 5 health posts and the topography and infrastructure in Ethiopia often make travel difficult. This is likely to have particularly been the case in SNNPR, which is Ethiopia’s most rural region. As part of an effort to reach targets set in the National Reproductive Health Strategy to expand access to LARCs, the Ethiopian FMOH has recently introduced an initiative to train select HEWs in both the insertion and removal of implants and intrauterine devices.^15^ This effort is especially relevant in light of our findings, which demonstrate that even when implant introduction efforts try to build in safeguards to ensure timely removals (deploying higher-level providers to the community level and referring women to higher-level facilities for removals), they may not be sufficient to fulfill the removal needs of all women, particularly those living in rural areas who cite transportation or distance to the facility as barriers to care. Other projects piloting the “task-shifting” of implant provision have also demonstrated effective insertion skills by providers,^16^^,^^17^ but information on removal services available to women through these efforts is still limited.

Married women and women with more education were less likely to have kept their implant for more than 3 years. Though women were not asked about the reasons for having their implants removed, these differences could be due to married women being more likely to remove their implant to have another child and those with more education better comprehending when and at what time point they should have their implant removed. Interaction models indicated that older women and women of other religions who had their implants inserted by HEWs were significantly more likely to be using them past 3 years. For older women, they may have completed their childbearing and not see the necessity of seeking removal; however, it is unclear if these women believe that the implant may prevent pregnancy for longer than 3 years.

The most common barriers to removal among respondents who kept their implant beyond 3 years were that they forgot, did not know the removal date, or faced transportation or distance barriers in returning to the facility. These barriers are reminiscent of some of the challenges to implant removal that have been documented in other contexts. For instance, a recent study in Ghana found that about half of women did not know that they could get their implant removed at a different health facility from the one where they had the implant inserted.^18^ After Implanon was introduced in South Africa, a study found that almost all women reported knowing that the implant should be removed after 3 years, but only two-thirds were told at insertion how long the implant can be used for and half were told when to return for removal. One woman specifically noted that she faced difficulties accessing removal services, first being told that the provider who originally inserted the implant was not available and being referred to another clinic, which subsequently told her she had to return to the same clinic where the implant was inserted for removal.^19^ Recommendations from the Indonesia Norplant experience were that programs introducing implants should plan to have removal services available from the time that insertions are first provided.^20^

The most common barriers to removal among respondents who had their implant beyond 3 years were forgetting, not knowing the removal date, or having impediments getting to the facility.

Strengths of this study were its large sample size and rigorous sampling process, which was implemented to gain a sample generalizable to Ethiopian women’s experience with 1-rod etonogestrel contraceptive implant insertion and removal by HEWs and other health care providers across the 4 regions of the country where the implant scale-up initiative was implemented. We surveyed women who had had an implant inserted 3–6 years prior so that we could assess their experience obtaining removals through the full recommended life of the implant. In addition, because we identified participants for this study from the facility registers, we presumed that women in this subset had not moved since the time of insertion. One might expect that their knowledge of where to access removal should be greater than users who have since relocated or migrated. Therefore, it is notable that this study population of users still faced challenges.

### Limitations

The study also had limitations, most notably challenges in obtaining accurate sampling frames of woredas that had started providing single-rod etonogestrel contraceptive implants at the community level, sampled sites that were inaccessible due to road conditions or a lack of eligible women that then required replacement, and security challenges in Amhara during the study period that curtailed data collection in that region and slightly reduced the precision of our estimates. As with any study collecting data retrospectively, participants’ responses may have been affected by recall bias. In this particular case, because Implanon is recommended to be used for up to 3 years, that may be the date that most women remembered, therefore we may be under- or overrepresenting the number of women who re-ported removing their implant within the 3 years duration. (Notably, respondents reporting using their implant for exactly 3 years constituted the largest proportion of our sample.) Finally, survey questions were limited to asking about any barriers to removal for each woman’s specific duration of use. Therefore, we were limited in our ability to assess a few relevant factors because they were not queried in the survey; these included asking whether women sought multiple removal attempts, if women who kept their implant beyond 3 years did so because they were continuing to rely on it for pregnancy prevention, and whether women who had their implants removed obtained removals through organized outreach activities at the health posts or at other health facilities.

## CONCLUSION

Given the dramatic increase in implant use globally over the past decade,^21^ ensuring access to timely removals is a responsibility of family planning programs guided by a rights-based approach to voluntary use of contraception.^22^ Although clinical data suggest that the effectiveness and safety of 1-rod etonogestrel contraceptive implants persist beyond 3 years, if a woman wants an implant removed (to become pregnant or for any other reason), not being able to obtain removal on demand is problematic and in conflict with principles of agency, autonomy, and empowerment. Access to removal services in Ethiopia has likely already improved in the nearly 4 years since this study was conducted. However, given efforts to promote task sharing of implant services at lower levels of health care systems worldwide, the Ethiopian experience is still important to document and learn from. Future efforts to increase access to implants may need to include innovative, locally appropriate mechanisms to remind women of the duration for which they have had their implant inserted to alert them of the recommended timeframe for removal as well as removal training for all health care providers who provide implant insertions.

## Notes

**Peer Reviewed**

**First published online:** August 25, 2020
